# New products

**DOI:** 10.1155/S1463924692000233

**Published:** 1992


Journal of Automatic Chemistry, Vol. 14, No. 3 (May-June 1992), pp. 107-111

### New products

### AA performance

The EDL System 2 is an electrodeless discharge lamp system which provides significantly higher spectral output than corresponding hollow cathode lamps. The system can be used in modulated or continuous modes with most Perkin-Elmer AA instrumentation. There are two major components: a dual-channel power supply with matched dual RF driver assemblies for simultaneous, independent operation of two EDLs and interchangeable lamp 'sleeves'. The RF power design integrates the RF power circuitry with the lamp driver assemblies, significantly reducing the cost of System 2 lamps and decreasing transmission power losses.

The system offers improved lamp life, signal-to-noise ratios, detection limits and precision, particularly for such difficult elements as arsenic and selenium.

*For further information contact Perkin-Elmer Limited, Post Office Lane, Beaconsfield, Buckinghamshire HP9 1QA, UK. Tel.: 0494 676161; fax 0494 678324.*

### X-ray microanalysis

The Microanalysis Group of Oxford Instruments, formerly known as Link Analytical, has introduced a new communications package called PC-Link that allows X-ray microanalysis or Link-exl users to access and apply third-party PC software, without tying-up electron microscope time. PC-Link is a rapid file-transfer system. However, it allows the PC to act as a second eXL terminal so data can be processed on the eXL before transfer, but without the user leaving the PC. This provides maximum convenience in preparing data for a report without the inconvenience of tying up the microscope.

Under PC-Link, images are converted to monochrome TIFF (Tag Image File Format) and text is converted to MS-Dos format. The system allows access to Ethernet via logical drive file names. If the PC is linked into an Ethernet ring, files can be transferred from the eXL to any other computer on the network.

*More information from Oxford Instruments, Halifax Road, High Wycombe, Buckinghamshire HP12 3SE, UK. Tel.: 0494 442255; Fax: 0494 524129.*

### Process wastewaters

Ionics UK Ltd have introduced the Water Policemen range of on-line monitors for continuous monitoring of process wastewaters. The Ionics range covers instruments for most wastewater parameters, including TOC, TC, TOD, COD and POC. Each model can be supplied either as a stand-alone instrument or as part of a more comprehensive control system. Everything from multi-stream samples to weather-proof enclosures are available.

Automating measurement of the wastewater's organic load can bring many benefits not merely in reduced pollution but in controlling product losses, in turn leading to greater process efficiency.

The most suitable analyser for a particular application can depend

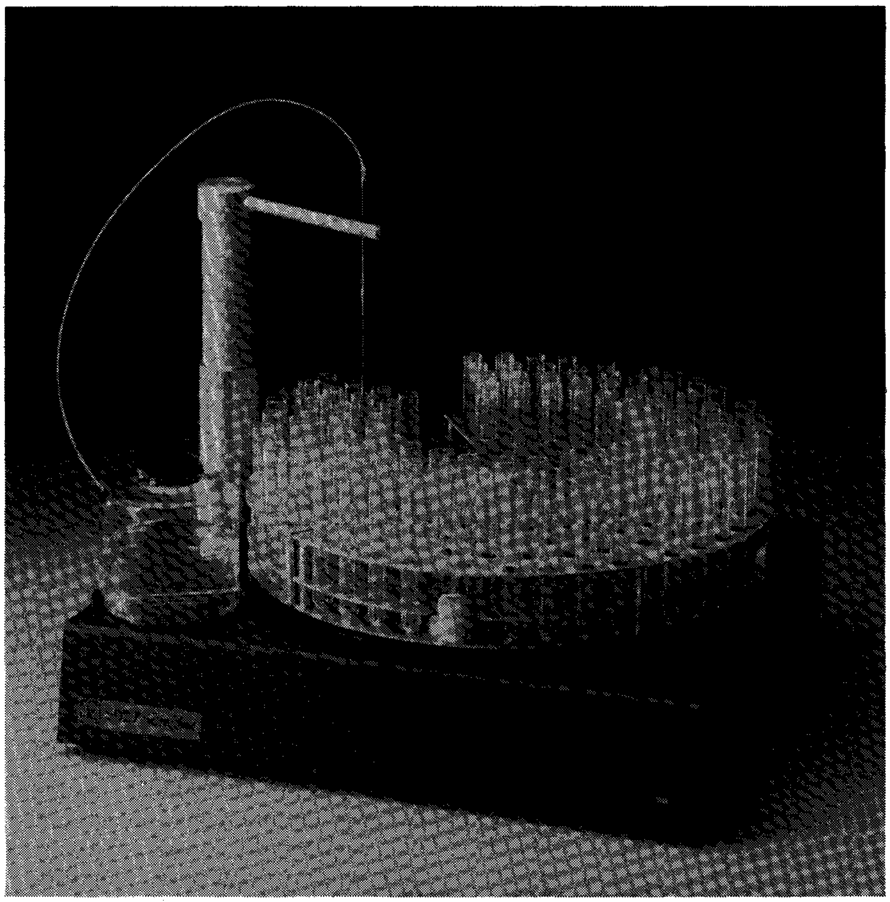

*The 5027 Sampler is designed for simple and safe automatic operation. The operator just loads the sample racks and positions the sample probe in the cup to start. The instrument software then assumes full control over the unit. Safety features include automatic release of probe and its arm if anything obstructs its movements. The 5027 sampler was designed to be used in a wide range of analytical systems – it is equipped with both TTL and BS 232C communication ports so it is easy to interface to a PC and integrate with other analytical systems. Three different turntables are available: a 32-position turntable which holds 100 ml cups; 120-position version for 4 ml cups; and a 120-position version designed for 12 ml tubes. More information from Tecator AB, Box 70, S-263 21 Höganäs, Sweden.*

0142-0453/92 $3.00 © 1992 Taylor & Francis Ltd.

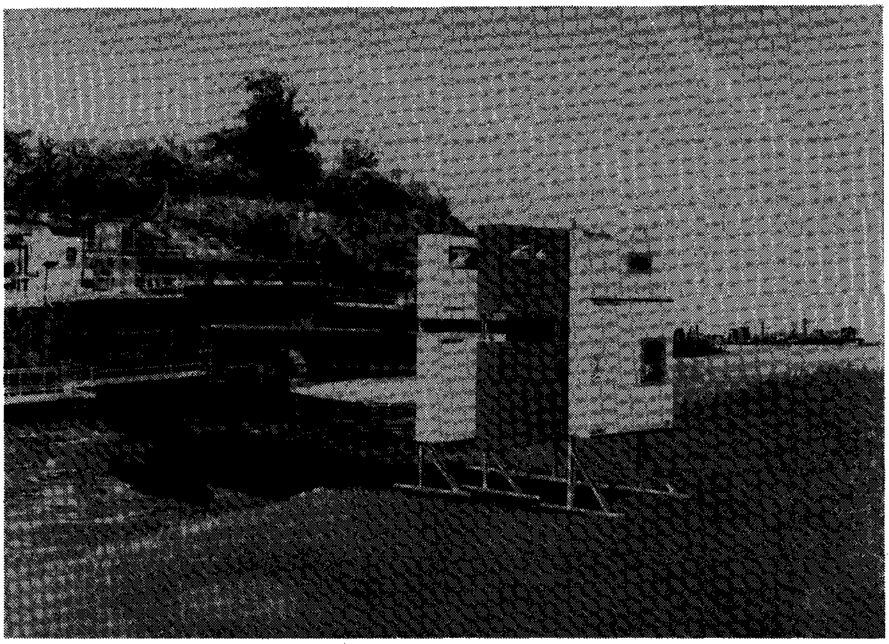

*The Ionics Water Policeman range for wastewaters – include TOC, TC, TOD, COD and POC.*

upon many factors – the manufacturer's processes, the chemicals involved and whether the monitoring application is a 'dirty-water' one where the chemicals are actually being discharged under consent directly into a river, or a cleanwater application where contamination is unlikely and monitoring is carried out purely to prove and maintain the plant's environmental integrity.

There can be so many reasons for choosing TOD over COD, TOC over TC, or vice versa and therefore Ionics work closely with companies to develop a monitoring system for their particular needs.

*Further details are available from Ionics UK Ltd, Carrington Business Park, Carrington, Urmston, Manchester M31 4DD, UK. Tel.: 061 776 4550; fax: 061 777 9630.*

### Drugs of abuse

Hewlett-Packard has published three new Application Notes, available from the company free of charge, which describe the analysis of, respectively, PCP ('angel dust') in urine; LSD in urine; and marijuana psychoactives in blood. HP GC/MS systems are used to confirm and quantify such common drugs of abuse with a sensitvity that exceeds the requirements of US regulatory authorities.

*Copies from Verena Haller, Hewlett-Packard SA, 150 Route du Nant-d'Avril, CH 1217 Meyrin (GE 2), Switzerland.*

### ICP spectrometers

The GBC Scientific Equipment's 950 series of ICP optical emission spectrophotometers which were launched in the UK at the end of 1991, is fully computer controlled, from plasma ignition to results print out. Operating parameters are monitored on the computer screen and controlled from the keyboard. A high-precision concentric nebulizer ensures high standards of analytical performance for aqueous and organic samples. A fully demountable, self-aligning torch, which features drop-in replacement glassware, simplifies maintenance and minimizes downtime.

The choice of optical systems includes simultaneous (polychromator), sequential (monochromator) or both modes of operation. The wavelength range extends from 160 to 820 nm, and all systems are fully thermostated for temperature stability.

*More information from GBC Scientific Equipment UK Ltd, 13 Frederick Sanger Road, The Surrey Research Park, Guildford, Surrey GU2 5YD, UK. Tel.: 0483 304988; fax: 0483 303071.*

### High solids analyser for flame AAS

GBC Scientific Equipment UK Ltd's HSA3000 High Solids Analyser, is an accessory for the GBC904, 905, 906 and 908 atomic absorption spectrophotometers. The HSA3000 is a sample-introduction system which relies on the injection of a small amount of sample into a continuous flow of rinse solution. This rinse solution ensures that the nebulizer and burner are continuously washed and are not blocked with salt. The small sample size results in a transient peak which is measured by the peak height or peak area mode. Precisions of around 2% and throughput of 50 samples per hour have been achieved with samples containing up to 30% dissolved salts.

Samples may be presented manually, or automatically, using the GBC FS3000 autosampler. The HSA3000 is controlled by the computer and software of the GBC 904, 905, 906 or 908 AAS.

*For further information contact GBC Scientific Equipment UK Ltd, 13 Frederick Sanger Road, The Surrey Research Park, Guildford, Surrey GU2 5YD, UK. Tel.: 0483 304988; fax: 0483 303071.*

### Enhanced calculations for reaction kinetics

Enhanced calculations of reaction times and capacities, are possible using the VIA204 Special Kinetics Module in conjunction with the Radiometer TitraLab Titration System. Reaction times needed to attain a given substrate conversion can be determined at up to four stages of a reaction. In addition, calculations of reagent consumption at four reaction times can be obtained.

Radiometer's TitraLab system uses the pH-stat technique to monitor reactions; this is ideal for studying reactions where hydrogen ions are consumed or released and involves determining the rate at which base or acid has to be added to maintain a constant pH value.

One of the most common applications of the TitraLab system is determining the neutralizing capacity and reaction times of antacids,

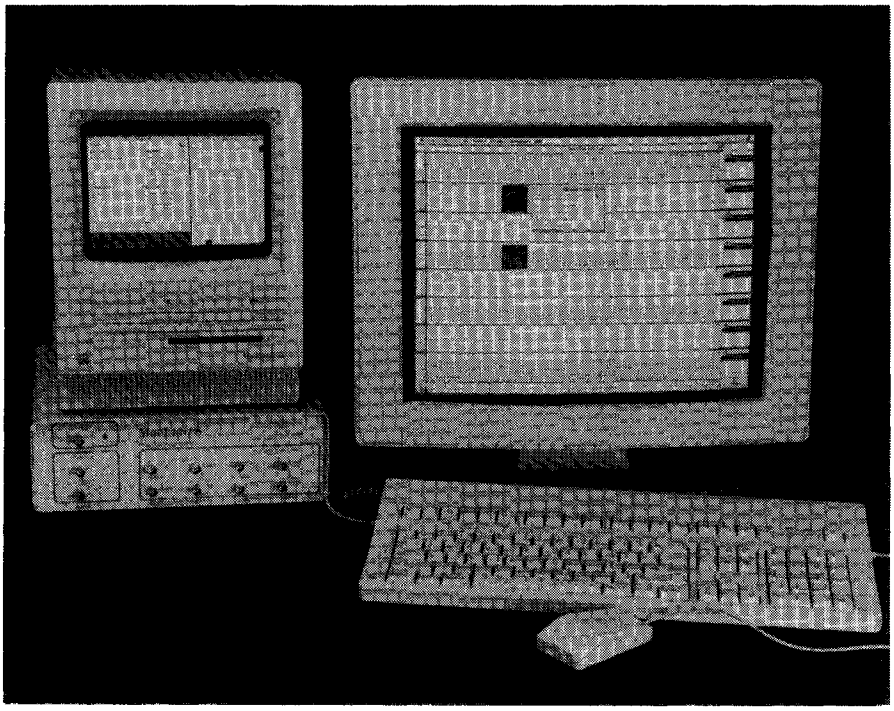

*The MacLab Division of ADInstruments, Inc. has introduced a 3·2 version of its CHART virtual chart recorder software for the Apple. CHART software provides all the functions of a high-end multi-channel chart recorder/polygraph, plus integration, statistical analysis, overlay and several post-experimental operations. Incoming data can be processed in real-time to provide on-the-fly computation of rate (in beats per minute or dV/dt), frequency (in Hz), event counting, means, cyclic maximum or minimum and minimum or maximum envelope tracking. Data can be recorded directly to disk or to system memory and can be collected in the background while the user analyses data from previous experiments, or runs other software in the foreground. Details from MacLab Division, ADInstruments, Inc., PO Box 845, Milford, Massachusetts 01757, USA.*

but enzyme activity assays also benefit for the enhanced calculation capabilities of the Special Kinetics Module. In this application, the slope of the reaction curve is calculated and expressed as a conversion rate in user-defined units. Statistics, including mean values and standard deviations of replicate analyses, are also available.

*Details from Radiometer, The Manor, Manor Royal, Crawley, West Sussex RH10 2PY, UK. Tel.: 0293 517599; fax: 0293 531597.*

### High specification Holter system

Oxford Medical have launched a high-performance version of Medilog OPTIMA. The product now features a full-specification Intel 386 microprocessor and a 210 Mbyte hard disc. High speed retrospective ECG analysis is provided on a wide range of tape types. Oxford tapes are analysed in about six mins, and Oxford solid-state recordings provide report pages on-screen in only a few seconds. The hard disc provides storage of up to six full two-channel 24 hour ECG tape recordings. A batch-processing feature allows analysis and printout of those recordings (or a number of solid-state recordings) whilst the operator is away from the system.

*Further details from Oxford Medical Ltd, 1 Kimber Road, Abingdon, Oxfordshire OX14 1BZ, UK. Tel.: 0235 33433; fax: 0235 34465.*

### HP laboratory robotics system

Hewlett-Packard's new laboratory robotics system, ORCA, is now available world-wide.

HP ORCA – the HP Optimized Robot for Chemical Analysis – is a flexible system designed to automate the complete sample analysis process. It meets the needs of laboratory automation development groups aiming to enhance the productivity of analytical applications. The system addresses the last area of the analytical process to benefit from automation: sample preparation.

The system, comprising a robotic arm, accessories and Microsoft Windows-based software, is used to move samples between various laboratory instruments and peripherals to perform complex sample preparation operations and analyses. It incorporates digital servo technology, harmonic drives and optical encoders to ensure precision and reliability.

The system fits easily on any standard laboratory bench, and its anthropomorphic design allows easy access to a wide range of laboratory equipment at virtually any orientation. The robotic arm has six degrees of freedom and travels horizontally along a 1 or 2 m precision rail. Interchangeable 'fingers' used by the robot are economical and provide broad-ranging functionality. The user can 'teach' the robotic arm specific movement patterns from the PC keyboard and a user-friendly joystick attachment.

Operation of the robot and the analytical instruments used in the application are controlled from an PH Vectra or other IBM-compatible personal computer, using HP Methods Development Software 2.0 (MDS 2.0). Using the Microsoft Windows graphical user interface, MDS 2.0 facilitates rapid development of analytical applications and is capable of executing multiple processes simultaneously.

For added flexibility and productivity, MDS 2.0 permits multitasking and data transfer to and from other Windows applications, including Microsoft Word and Excel.

The HP ORCA system can work with instruments and peripherals from many manufacturers – any instrument that can communicate with the HP Vectra PC or other host computer through industry-standard HP-IB, IEEE-488 or RS-232C interfaces can be incorporated into the HP ORCA robotics set-up.

*Enquiries to Verena Haller, Hewlett-Packard SA, 150 Route du Nant-d'Avril, CH 1217 Meyrin (GE 2), Switzerland.*

### Polarimeter

Optical Activity Ltd's high-performance polarimeter – the PolAAr 2001 breaks new ground with the ability to measure dark samples, absorbing up to 99·9% (3·00D) of the source beam, to a high accuracy and many other features. These include six measurement scales and calibration standards traceable back to International Standards Organizations.

Designed to give high accuracy results (to within 0·001° angular rotation), the PolAAr 2001 fulfils the needs of both research and process control analyst.

The instrument has six measurement ranges including a user-programmable scale. Each is selected through the touch button keypad. Meaurements are made at the internationally standard 589·44 nm (sodium yellow) wavelength. Dual wavelength versions are also available. The principal alternative wavelength is normally 546 nm (mercury green), but any wavelength between 400 nm and 900 nm can be selected. The calibration standards used in manufacuture are traceable back to the National Physical Laboratory and the German Standards Insititute – Physikalisch Technische Bundesaustalt.

The PolAAr 2001 accepts all internationally standard sample cells including flow-through and microcells. The ventilated sample compartment has been designed to ensure that the sample temperature never rises by more than 3 °C above ambient, even over long measurement times.

A multiplier factor from 0·02 to 9·99 can be applied to all measurements to simulate changes in sample pathlength.

Results complete with either automatically or manually generated sample number are output by dual RS232 sockets to a printer or computer. They are simultaneously displayed on the built-in LCD readout on the instrument's front cover.

*Further information from Optical Activity, Bury Road Industrial Estate, Ramsey, Huntingdon PE17 1NA, UK. Tel.: 0487 813913; fax: 0487 812789.*

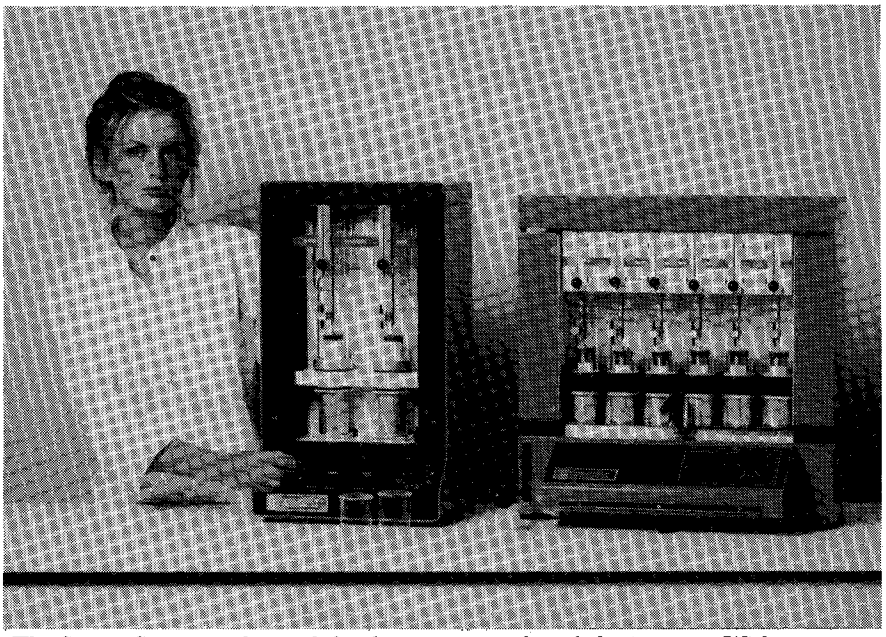

*The Soxtex System is designed for determination of total fat in meat. With a two-step extraction technique, Soxtec reduces the extraction time for fat determination in meat about four times, compared to the traditional Soxhlet reference method. Costs are reduced by about 70% due to the lower volume required for each fat extraction and a special solvent recovery feature. Finally the risk of solvent vapour explosions is completely elminated. Additional information from Tecator AB, Box 70, S-263 21 Höganäs, Sweden.*

### Managing and analysing technical data

Kingfisher is a software tool for UNIX workstations – it offers analytical and spectroscopic systems users a creative solution to their combined needs for a database and graphical data analysis facilities.

A particularly useful application of Kingfisher is that it allows a laboratory to link up equipment from a variety of techniques, for example, mass spectroscopy, gas chromatography, infra-red chromatography scintillation techniques, etc. This is not only more effective for storing and analysing data, including cross-technique analyses, it also takes the processing burden away from the analysis equipment, leading to much higher utilizations of these costly pieces of apparatus.

Traditionally, users of relational databases have either been obliged to learn and use a conventional Relational Query Language, which is cumbersome and tedious, or build database applications, which can be a slow and inflexible process. Instead, Kingfisher conceals the Query Language by adopting a highly intuitive graphical user interface – users interact with a visual representation of their databases and associated tables arranged on a screen workspace.

Retrieval of data, setting relational conditions and applying mathematical functions to the data are accomplished using Iconstyle 'tools' which are 'dropped' on to the workspace. This, together with extensive use of OSF-Motif GUI technology, yields a user interface which is extremely natural, far more productive than a conventional Query Language interface, and obviates the needs for extensive training.

Analysis and visualization of data is traditionally handled by distinct software packages that are separate from the database. In Kingfisher these facilities are tightly integrated with the database functions. Retrieved data is presented directly in graph form in a separate display window.

Users can view their data using a wide range of graph styles, from 2D line graphs through scatter and polar charts and on to 3D waterfall displays. Overlays showing items such as means, standard deviations and fitted curves can be applied and automatically recalculated whenever new data is displayed.

Kingfisher includes facilities for working with data graphically; for example, zooming down on selected regions, a ruler for measuring distances and finding the equation of a line, and a data editor for examining and changing data, point by point.

*Details from Leading Technology, 11 Grosvenor Place, London SW1X 7HH. Tel.: 071 235 5498; fax: 071 823 1660.*

### Sequential plasma emission spectrometer

An eight-page colour brochure for the AtomScan 16 plasma emission spectrometer is now available from Thermo Jarrell Ash Corporation. The AtomScan 16 is a fully automatic system which incorporates computer selectable torch power levels; the programmable gas flows; low argon flow/low R.F. power for routine samples and high flow/high R.F. power for difficult samples. An autotuned, crystal-controlled R.F. generator permits determinations of elements in both organic and aqueous samples. The galvanometer grating drive is covered by a 15-year warranty. A powerful AT compatible data system and ThermoSPEC software facilitate both methods development and elemental analysis and it comes with Multiquant, a protocol for performing rapid screening of sample to determine approximate compositions.

*Copies from Thermo Jarrell Ash Corporation, 8E Forge Parkway, Franklin, Massachusetts 02038, USA.*

### On-line degasser for HPLC solvents

An on-line degasser that removes dissolved gases from solvents used in high performance liquid chromatography (HPLC) analysis has been announced by Helwett-Packard. Developed at the company's Waldbronn Analytical Division in Germany, the new unit can degas up to four different solvents simultaneously using a continuous vacuum process.

Dissolved oxygen forms a UV-absorbing complex with many solvents used in HPLC, adversely affecting the performance of UV/visible and fluorescence detectors. Dissolved gases also affect the performance detectors. Dissolved gases also affect the performance of HPLC pumps, resulting in unstable baselines or unreproducible retention times. The HP 1050 Degasser keeps dissolved gases at a constant low level, preventing baseline drift and ensuring optimum detector and pump performance.

Degassing efficiency is evaluated by measuring the oxygen concentration in the degassed solvent and comparing before and after sensitivity levels with detectors sensitive to gas bubbles. The HP 1050 Degasser maintains an oxygen concentration level of 1·5 parts-per-million at a flow rate of 10 ml/min per channel.

The unit operates by passing solvents through special plastic membrane tubes that are permeable to gas but not to liquid. The tubes are housed in four separate vacuum chambers. The pressure difference between the inside and the outside of the membrane caused continuous degassing of the solvent.

As a stand-alone module, the HP 1050 Series On-Line Degasser can be used in conjunction with any HPLC pump or HPLC system. When used with the PH 1050 Series modular HPLC system, it stacks between other modules, so taking up no extra laboratory bench space.

*Enquiries to Verena Haller, Hewlett-Packard SA, 150 Route du Nant-d'Avril, CH 1217 Meyrin (GE 2), Switzerland.*

### Densimeter

Increasing demands for an inexpensive densimeter that accurately measures specific gravity of both liquids and solids prompted A&D Instruments to launch the SD-120L. The SD-120L is a highly precise electronic densimeter that can calculate specific gravity very quickly, with a resolution of 0·0001 and measuring tolerance of ±0·0002.

Specific gravity is measured for solids by means of the Archimedean principle – typically by comparing the weights of a sample in air and in water (although other solutions can be substituted, if preferred). Assessments can equally be made in the liquid mode, by comparing a standard glass sample's weight in air and in 50 cc of the liquid under test. Testing in the liquid mode, however, does require the optional liquid gravity kit.

*Further information from A&D Instruments, Abingdon Science Park, Abingdon, Oxfordshire OX14 3YS, UK. Tel.: 0235 550420; fax: 0235 550485.*

### Water analysis

Perkin-Elmer now offers two cassette kits designed for water and environmental analyses with the Lambda 2 UV/VIS spectrometer. The first is a cassette preprogrammed for the analysis of 20 different water constituent, both manually and with automatic sipper operation. The second package consists of two cassettes with a total of 113 preprogrammed methods of use with Merck Spectroquant reagent kits. These kits are widely used in the control of process waters and effluents. The Lambda 2 UV/VIS spectrometer is designed for routine and automated analyses and has a wavelength range between 190 and 110 nm. Prepared basic methods for spectra recording, wavelength programming, kinetics and quantitative analysis can be user-modified and stored as ready-to-use methods – a principle which is particularly suited to routine water analysis.

*For further information contact Perkin-Elmer Limited, Maxwell Road, Beaconsfield, Buckinghamshire HP9 1QA, UK.*

